# Cyclophilin D in Mitochondrial Dysfunction: A Key Player in Neurodegeneration?

**DOI:** 10.3390/biom13081265

**Published:** 2023-08-18

**Authors:** Gabriele Coluccino, Valentina Pia Muraca, Alessandra Corazza, Giovanna Lippe

**Affiliations:** Department of Medicine (DAME), University of Udine, 33100 Udine, Italy; muraca.valentinapia@spes.uniud.it (V.P.M.); alessandra.corazza@uniud.it (A.C.)

**Keywords:** Cyclophilin D (CyPD), mitochondrial permeability transition (mPTP), Alzheimer’s disease (AD), Parkinson’s disease (PD), mitochondria, neurodegeneration

## Abstract

Mitochondrial dysfunction plays a pivotal role in numerous complex diseases. Understanding the molecular mechanisms by which the “powerhouse of the cell” turns into the “factory of death” is an exciting yet challenging task that can unveil new therapeutic targets. The mitochondrial matrix protein CyPD is a peptidylprolyl *cis*-*trans* isomerase involved in the regulation of the permeability transition pore (mPTP). The mPTP is a multi-conductance channel in the inner mitochondrial membrane whose dysregulated opening can ultimately lead to cell death and whose involvement in pathology has been extensively documented over the past few decades. Moreover, several mPTP-independent CyPD interactions have been identified, indicating that CyPD could be involved in the fine regulation of several biochemical pathways. To further enrich the picture, CyPD undergoes several post-translational modifications that regulate both its activity and interaction with its clients. Here, we will dissect what is currently known about CyPD and critically review the most recent literature about its involvement in neurodegenerative disorders, focusing on Alzheimer’s Disease and Parkinson’s Disease, supporting the notion that CyPD could serve as a promising therapeutic target for the treatment of such conditions. Notably, significant efforts have been made to develop CyPD-specific inhibitors, which hold promise for the treatment of such complex disorders.

## 1. Introduction

With the global increase in life expectancy and population, the prevalence of age-associated diseases has become a significant challenge in recent years. Among these diseases, neurodegenerative disorders stand out as a major social and health burden, and their rapid and alarming rise underscores the need for new therapeutic interventions. Aging is widely recognized as the most significant risk factor for the development of neurodegenerative disorders, including Alzheimer’s disease (AD) and Parkinson’s disease (PD) [1]. Neurodegenerative diseases can be categorized into two main types: early-onset familial cases, which are rare and account for 5% to 10% of all patients, and late-onset sporadic cases, which are more common and account for 90% to 95% of all patients. Familial cases typically manifest before the age of 65 and are caused by mutations in specific genes, such as α-synuclein, Pink1, and Parkin in PD, and amyloid precursor protein (APP) and presenilin (PS) in AD. On the other hand, sporadic cases are much more complex and result from a combination of genetic and environmental factors [2].

Neurons are highly specialized cells that require a substantial amount of energy to function properly. Therefore, it is not surprising that mitochondrial dysfunction has emerged as one of the hallmarks of dysfunctional neurons. However, the exact relationship between mitochondrial and neuronal dysfunction is not fully understood, and it remains unclear whether mitochondrial dysfunction is a concomitant cause or an indirect effect of neurodegeneration.

This review focuses on the mitochondrial protein Cyclophilin D (CyPD) and its involvement in neurodegeneration, with a particular focus on the two most spread neurodegenerative disorders, i.e., AD and PD.

## 2. The Superfamily of Peptidylprolyl *cis-trans* Isomerases

Peptidylprolyl *cis*-*trans* isomerases (PPIases), or rotamases, are conserved enzymes (EC 5.2.1.8) able to catalyze the *cis*-to-*trans* isomerization of proline residues, a rate-limiting step in protein folding [3]. The presence of an enzyme with PPIase activity was first described in 1984 by Fischer et al. [4] from porcine kidney extracts. In the same year, Handschumacher et al. [5] described the first natural receptor of the immunosuppressant Cyclosporine A (CsA) in the cytoplasm of bovine thymocytes and named it Cyclophilin. However, it was only in 1989 that the N-terminal sequencing of porcine PPIase revealed that the PPIase and Cyclophilin were the same protein [6]. Nowadays, the superfamily of PPIAses is divided into immunophilins, comprising Cyclophilins (CyPs) and FK506-binding proteins (FBKPs), and Parvulins. Parvulins, CyPs, and FKBPs constitute three distinct families of structurally unrelated PPIases whose members can be found in all cellular and extracellular compartments [7]. Moreover, it has been shown that CyPs, FKBPs, and Parvulins have different substrate specificities. In particular, CyPs and FBKPs efficiently recognize the AXPF motif, with CyPs more efficient on small hydrophobic residues (such as Ala) and FBKPs more specific on bigger residues (such as Phe or Leu) [7]. Parvulins are instead specific on the *cis*-*trans* isomerization of phosphorylated motifs such as pSer-Pro or pThr-Pro [7,8].

The enzymatic mechanism of the isomerization has not been completely established yet, and several models have been proposed [9]. It should be noted that the effective importance of the PPIase activity in vivo has been questioned, and some suggest that it could be less relevant than expected [10]. Indeed, in proteins, the *cis* isomers of the Xaa-Pro motifs represent only about 5–6% of the population [11], and several PPIAse-associated functions in the physiological context do not depend on their enzymatic activity [10]. In this scenario, PPIases would serve just as scaffolds or interaction hubs, and exert their function regardless of their enzymatic activity [10], which in several cases have been shown to be dispensable.

## 3. Generalities of CyPD

Cyclophilins were initially described as CsA-sensitive PPIases [12]. Today, there are at least 17 genes (and a plethora of pseudogenes) annotated as Cyclophilins in the human genome (Table 1) that differ from one another in terms of subcellular localization, function, and interaction clients. Indeed, while it was originally thought that CyPs were involved only in protein folding [3,4], it is now widely accepted that they exert many different roles in a wide variety of cellular processes, such as gene transcription [13], signal transduction, RNA maturation [14], host-pathogen interaction [15], and chemotaxis [16], not necessarily dependently on their PPIase activity.

CyPD, which is encoded by the *ppif* gene, is the only CyP present in the mitochondrial matrix. It should be noted that the nomenclature of CyPD and the relative *ppif* gene can generate some confusion. The *ppif* gene was first discovered by Bergsman et al. in 1991 [17], who named it *hCyP3*; the protein was then referred to as CyP3. In the subsequent years, the usage of the term CyPD referring to the mammalian mitochondrial cyclophilin became widespread, as reported by Woodfield et al. already in 1997 [18]. However, the HUGO gene nomenclature annotated improperly the gene encoding the cytosolic CyP-40 as *ppid* (peptidylprolyl *cis*-*trans* isomerases D) while the gene encoding for the mitochondrial CyPD was annotated as *ppif* (peptidylprolyl *cis*-*trans* isomerases F) [19,20]. This ambiguity can occasionally lead to confusion between CyP-40, which is also sometimes also referred to as CyPD, and the mitochondrial CyPD. Here, in line with most of the literature, CyPD will always refer to the gene product from *ppif*.

**Table 1:** Human Cyclophilins. The human genome encodes for 17 different genes annotated as CyPs. Based on GeneCards [21] and Uniprot [22] data, the table shows for each of them the chromosomal localization, the name(s) of the respective proteins, their lengths, and subcellular localizations.

Gene	Chromosome	Protein Name(s)	Length (AA)	Subcellular Localization
*ppia*	7p13	CyPA	165	Cytoplasm, Nucleus, ECM
*ppib*	15q22.31	CyPB	216	ER Lumen
*ppic*	5q23.2	CyPC	212	Cytoplasm
*ppid*	4q32.1	CyP-40, CyPD	370	Cytoplasm, Nucleus
*ppie*	1p34.2	CyP-33, CyPE	301	Nucleus
*ppif*	10q22.3	CyPD, CyP3, CyP-M, CyPF	207	Mitochondrial Matrix
*ppig*	2q31.1	SRCyP, SCAF10	754	Nucleus, Nuclear Speckles
*ppih*	1p34.2	CyP-20, CyPH, USA-CyP	177	Cytoplasm, Nuclear Speckles
*ppil1*	6p21.2	CYPL1, hCyPX, CGI-124	166	Nucleus
*ppil2*	22q11.21	CyP-60, CyC4, UBOX7	520	Nucleus
*ppil3*	2q33.1	CyPJ	161	Nucleus
*ppil4*	6q25.1	PPIL4, HDCME13P	492	Nucleus
*ppil6*	6q21	PPIL6, RSPH12	311	Cytoplasm
*ppwd1*	5q12.3	PPWD1	646	Nucleus
*ranbp2*	2q13	RANBP2, NUP358	3224	Nucleus
*cwc27*	5q12.3	CWC27, SDCCAG10	472	Nucleus
*nktr*	3p22.1	CyPNK, P104	1462	Cell Membrane

**Figure 1:**
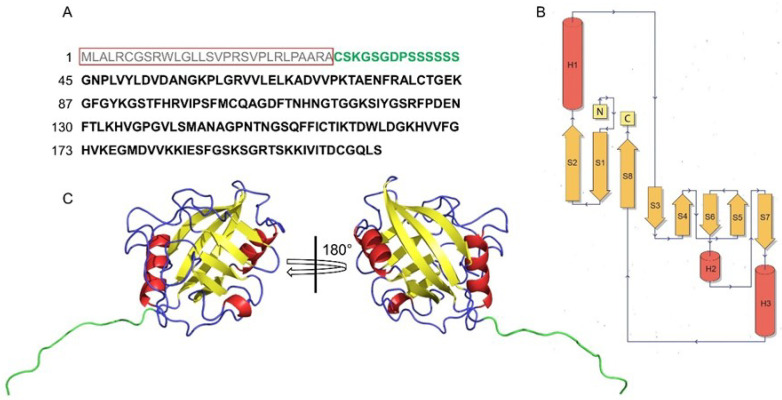
The CLD domain of human CyPD. (**A**) One-letter code amino acidic sequence of CyPD. The red box highlights the MTS, which is cleaved by matrix signal peptidases and therefore is not part of the mature protein. Residues in green are missing in all the crystallographic structures deposited so far in the PDB. (**B**) Secondary structure topology diagram of the CLD domain. Helices (H1–H3) are in red, sheets (S1–S8) are in yellow. (**C**) Two views of the cartoon-represented FL-CyPD predicted by AlphaFold (AF-P30405-F1), colored according to the secondary structure. Left: active site side. Right: back face side Residues in light blue correspond to the ones in (**A**).

The numbering of CyPD residues (Table 2) can be quite confusing as well and has led to misunderstandings in the field. The human *ppif* gene encodes for a 207 residues protein whose 29-residues mitochondrial targeting sequence (MTS) is cleaved by the matrix peptidases upon entering the mitochondrion [23] (Figure 1A). Therefore, the mature CyPD consists of 178 residues. To further complicate the picture, crystal structures are based on a truncated form of CyPD lacking the first 14 residues (see below) and therefore consisting of 164 amino acids (Figure 1A). Thus, it has happened that the same residue has been numbered in literature in three different ways depending on the numbering system adopted. Here, we will number the residues according to the mature form of CyPD, reporting in brackets the numbering that takes the MTS into account when needed.

CyPD was first identified in 1992 by Connern and Halestrap [24], who described the presence of a PPIase distinct from CyPA in the mitochondrial matrix of rat livers.

It is now well-established that CyPD is a key protein in the mitochondrial proteome [25]. Indeed, it has been shown to interact with numerous diverse protein partners, including F-ATP synthase [26,27,28,29], the adenine nucleotide transporter (ANT) [30], the protein kinases GSK-3β [31] and Akt2 [32], p53 [33,34,35], the molecular chaperone TRAP-1 [36], complex III of the electron transport chain [37], and Bcl-2 [38] among others. Thus, it is not surprising that CyPD exerts many diverse biological functions in the mitochondrial homeostasis. For example, it has been shown to regulate the dynamic formation of mitochondrial supramolecular complexes such as the respirasome [37] and ATP Synthasome [39], although it should be noted that the existence of the latter is still a matter of controversy due to the large conformational changes that the ANT needs to adopt during its function [40,41]. Moreover, it was shown that it indirectly regulates the acetylation of mitochondrial proteins [42]. However, the best-characterized function of CyPD is the regulation of the mitochondrial permeability transition pore (mPTP). The mPTP is a calcium-dependent unselective channel in the inner mitochondrial membrane involved in calcium and ROS homeostasis, whose long-last opening leads to mitochondrial dysfunction and is associated with pathology [43]. For example, it has been associated with the detrimental effects of ischemia/reperfusion (I/R) of several organs, particularly the heart [44]. The initial observation that CsA inhibits pore opening [45] led to the subsequent observation that CyPD plays a crucial role as a regulator of the channel [46], even though it is not a part of its molecular structure [47]. Current evidence indicates that the mPTP is primarily formed by F-ATP synthase [28,29], although the involvement of ANT has not been excluded [43]. Indeed, it has been shown that CyPD interacts with the lateral stalk of F-ATP synthase [26], particularly with subunit OSCP (oligomycin sensitivity-conferring protein) [27], and that this interaction is associated with the opening of mPTP [27]. More details on the mPTP will be reported in a dedicated paragraph (Section 6).

## 4. Structural Features of CyPD

The first structure of a CyP was determined by two groups in 1991 [48,49], who independently resolved the crystal structure of CyPA. In fact, at that time, due to the great interest in the immunosuppressant properties of CsA, significant effort was put into the structural characterization of the CyPA:CsA system. Indeed, in less than two years four independent labs published about 10 structures of free and CsA-bound CyPA [50], resolving them by both NMR and crystallography. Subsequent work showed that all CyP structures shared the CLD domain [51], whose architecture is well conserved throughout evolution [51,52]. Mitochondrial CyPD structure was resolved only in 2005 [53], almost 15 years later than CyPA, while the crystal structure of CyPD:CsA complex was determined in 2007 [54]. Since then, more than 60 structures of the human CyPD have been deposited on the PDB database [55], and they are all derived from crystallography studies. It should be noted that all the CyPD structures deposited so far are based on the work of Schlatter et al. [53] and therefore they are all truncated forms of CyPD, which lack the first 14 residues (Figure 1A) and carry a lysine-to-isoleucine mutation at position 146 (133 in the crystal). So far, the only structure available of the complete mature wild-type CyPD (FL-CyPD) is the one predicted by AlphaFold [56,57] (Figure 1C), which is highly similar to the crystallographic one (RMSD 0.2–0.3 Å).

The CLD domain consists of a β-barrel formed by 8 antiparallel β-sheets that form a compact hydrophobic core [48] connected by several loops and 3 helical elements (Figure 1C): the two α-helices H1 and H3, that confine the barrel at opposite sides, and the small H2, a single helical turn located between the strands S6 and S7 also reported [54] as a 3_10_ helix (Figure 1C).

**Figure 2:**
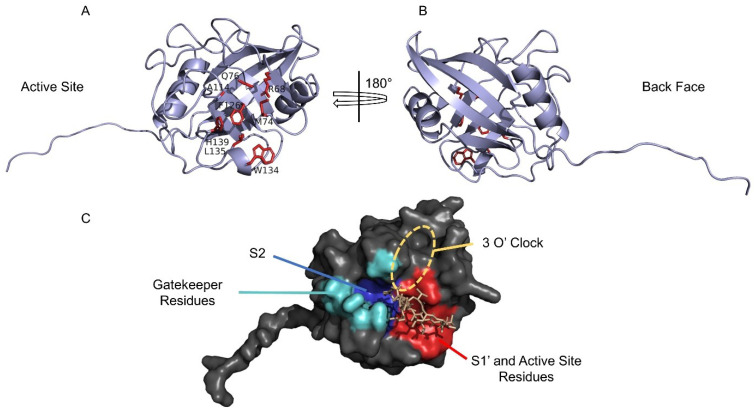
The active site and the back face of CyPD. (**A**–**C**) Different representations of the AlphaFold structure of CyPD. (**A**) The active site of CyPD includes a mixture of polar and non-polar highly conserved residues (sticks representation of sidechains, red). (**B**) The back face of CyPD is an underestimated site of protein-protein interactions. (**C**) The active site side includes several pockets. S1′ (red) and S2 (blue) are involved in the interaction with the substrate. The S1′ pocket, which accommodates the proline of the substrate, is surrounded by the active site residue (in red) and mediates the interaction with cyclosporine A. The S2 pocket, which is relatively non-specific, is surrounded by poorly conserved “gatekeeper residues” (cyan), which control the pocket and are therefore involved in substrate specificity. The “3 O’clock” pocket is a novel putative isoform-specific synthetic-inhibitor interaction site.

CyPD structure can be roughly divided into the active site side and the opposite face, the so-called “back face” (Figure 1C and Figure 2A,B). The back face of CyPD has been found to be a site of protein-protein interactions [52], further validating the idea of CyPs not only as PPIases but also, if not mostly, as interaction hubs.

The active site of CyPs (Figure 2A) comprises highly conserved residues: these include the catalytic arginine Arg68 (*R97*) and a mixture of non-polar and polar residues, i.e., Met74 (*M103*), Gln76 (*Q105*), Ala114 (*A143*), Phe126 (*F155*), Trp134 (*W163*), Leu135 (*L164*), and His139 (*H168*). Altogether, they form an extensive binding surface measuring ~10 Å along the R68-H139 axis and ~15 Å along the W134-A114 axis [52]. It has also been observed that W134, the only tryptophan present in CyPD, is required for CsA binding and that this is abrogated by all other natural variants occurring at this position, with the exception of histidine [52]. Careful analysis of the substrate-binding surface reveals that it is formed by two different pockets (Figure 2C), the S1′ pocket, comprising the highly conserved proline-binding residues, and the S2 pocket, which mediates the interactions with substrate residues P2 and P3 (relative to the substrate proline P1) [52]. The CyPD S1′ pocket is formed by Phe126 (*F155*) at its base and Phe73 (*F102*), Met74 (*M103*), Leu135 (*L164*), and His139 (*H168*) at its sides [52]. On the other hand, the S2 pocket surface is formed by the main-chain atoms of the S5-S6 loop, including mostly the residues between Met113 (*M142*) and Gln124 (*Q153*), making it relatively uniform across CyPs. The pocket surface is critically guarded by a set of gatekeeper residues (Figure 2C), whose sidechains control the access to the active site and therefore determine substrate specificity [52]. This set of residues is less conserved and would explain the different substrate specificities among all CyPs. For CyPD, the gatekeeper residues include Thr86 (*T115*), Ser94 (*S123*), Arg95 (*R124*), Ala116 (*A145*), Thr120 (*T149*), Ser123 (*S152*), and Gln124 (*Q153*) [52].

Lastly, the design of a novel CyPs inhibitor led De Simone et al. to describe an additional pocket perpendicular to the active site, called the “3 O’clock” pocket [58]. The 3 O’clock pocket is formed by residues not involved in substrate binding and therefore poorly conserved between different CyPs, which would represent a good candidate for isoform-specific CyPs inhibitors [58].

## 5. Cyclosporine A

Cyclosporine A (CsA) is a cyclic undecapeptide produced by the ascomycete *Tolypocladium inflatum*. Ever since the discovery of its immunosuppressive activity, first reported in 1976 by Borel et al. [59], it has been used to prevent the rejection of transplanted organs. The immunosuppressive function of CsA specifically involves the suppression of the activation of T cells, which starts with its binding to the cytosolic Cyclophilin A (CyPA) in T cells [60]. The CyPA:CsA complex binds and inhibits in a PPIase-independent manner the phosphatase activity of calcineurin which is required for the activation and nuclear translocation of NF-AT, a transcription factor that activates the transcription of cytokine genes, including IL-2 and IL-4 [60].

CsA has a high hydrophobic character, due to the N-methylation along the protein backbone of 7 residues out of 11 [61]; only a single hydroxylic group is present [61]. It was observed that in apolar (chloroform) [62] and polar (methanol, DMSO) [63] solvents CsA adopts a twisted β-sheet conformation with three intramolecular hydrogen bonds, similar to what is observed in the crystal structure [62]. The solution structure of the CyPA-bound CsA [50,64] revealed remarkable differences with free CsA, as it adopts no regular secondary structure and contains no intramolecular hydrogen bonds [61]. On the other hand, it has been shown that CsA-bound CyPA [65] and CyPD [54] are very similar to their CsA-free counterparts, i.e., do not undergo major conformational changes upon binding. Moreover, the CyPA:CsA and CyPD:CsA complexes have the same geometry (RMSD of 0.5 Å), in which CsA binds the active site of CyP [54]. The mechanism of PPIase inhibition by CsA has been explained in terms of the twisted-amide model and catalysis through distortion [12].

Although Cyclophilins were first defined as intracellular receptors of CsA [12], it should be noted that it was recently demonstrated that not all the proteins annotated as CyPs in the human genome are able to bind CsA [52]. In particular, PPIL2, PPIL6, RANBP2, and SDCCAG-10, all lacking the catalytic tryptophan, do not display either Cyclosporin binding or PPIase activity [52].

Independent data revealed that the affinity of CsA towards CyPs is in the nanomolar range. Specifically, the $K_{i}$ was determined to be in the 10–200 nM range [66], while the $K_{D}$ was found between 6.8 nM and 588 nM [52]. For CyPD, the $K_{i}$ determined with isothermal calorimetry is 6.7 nM [67], while the $K_{D}$ determined by fluorescence analysis was found 12.5 nM [53].

## 6. The Permeability Transition Pore

As stated above, CypD is the master regulator of the mitochondrial permeability transition pore (mPTP), an unselective, high-conductance channel, whose opening is activated by calcium, oxidative stress, and membrane depolarization [68]. The channel exhibits a maximal conductance of up to 1.3 nS and a variety of subconductance states with variable durations [68]. The consequences of PTP opening depend on the open time of individual pores (channel kinetics) and the number of open pores at any given time (population dynamics) [68]. The high conductance pore, when fully open, allows the passage of large solutes, with MW up to 1.5 KDa [69]. Extensive and prolonged mPTP activation triggers the collapse of the mitochondrial protonmotive force (pmf), thus inhibiting the oxidative phosphorylation, and the flood of a mitochondrial matrix with cytosolic solutes, leading to mitochondrial swelling and eventually rupture of the outer mitochondrial membrane. This latter event may cause cell death by necrosis [70] or the release of proapoptotic proteins, thereby inducing apoptosis or similar processes [71].

CyPD modulates the mPTP by decreasing the Ca^2+^ load required for pore opening, an effect that is prevented in the presence of CsA [46]. Several laboratories successfully created CyPD-null (*Ppif*^−^/^−^) mice [47,70,72], which showed no overt phenotype and no noticeable changes in mitochondrial function. As expected, the mPTP in *Ppif*^−^/^−^ mitochondria was desensitized to Ca^2+^ and was insensitive to CsA. Importantly, largely through the use of *Ppif*^−^/^−^ mice, a wide variety of murine models of human degenerative diseases were created that allowed to demonstrate the involvement of mPTP misregulation in several pathologies [73,74,75,76,77,78,79,80].

Although the molecular identity of the mPTP is a long-standing mystery, recent efforts have made it possible to obtain a definitive model of the pore components. The original observation [81] that PTP opening was affected by the selective inhibitors of the adenine nucleotide translocator (ANT), atractylate (ATR, which favors PTP opening), and bongkrekate (BKA, which favors PTP closure) suggested the involvement of the ANT in mPTP formation. An early hypothesis viewed the mPTP as a multiprotein complex including the core constituents ANT and voltage-dependent anion channel (VDAC) associated with hexokinase 2 and CyPD acting as modulators [82]. However, this model did not stand the test of genetics, as reviewed by Bernardi et al. [83]. After extensive debate and experimental work [84,85,86,87], the most recent model proposes a dual nature of mPTP formation, one involving F-ATP synthase as the leading mPTP pathway and the other involving the ANT [43]. This is based on the evidence that in the absence of a fully assembled F-ATP synthase, a small CsA- and BKA-sensitive channel still forms [88].

**Figure 3:**
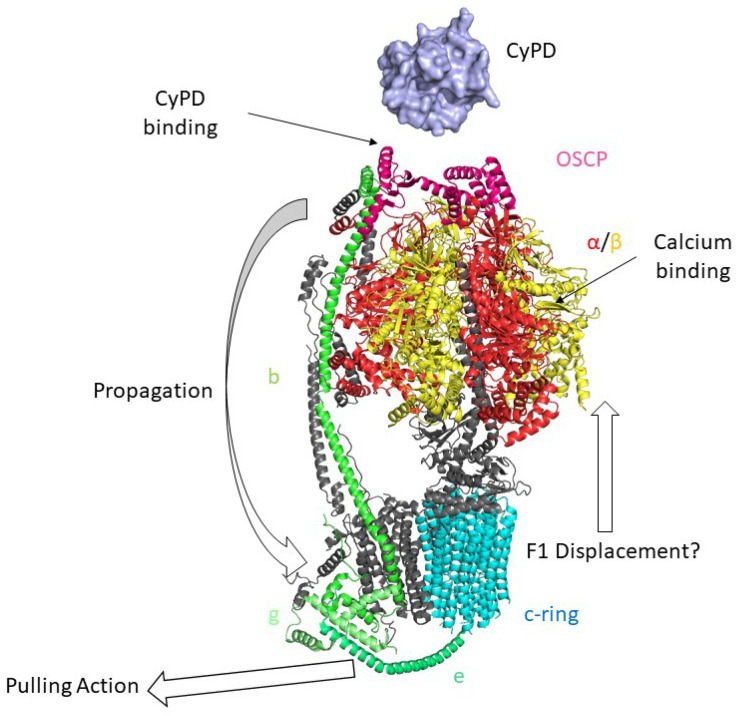
Mammalian monomeric F-ATP Synthase and the “Death Finger” Hypothesis of mPTP formation. Cryo-EM structure of F-ATP Synthase is shown (PDB ID: 6TT7 [101]). According to the death finger hypothesis, calcium binding to the α (red)/β (yellow) subunits induces conformational changes that are transmitted from OSCP (pink), the binding site of CyPD (light blue, PDB ID: 2Z6W [54]) to the lateral stalk of F-ATP synthase (subunit b in light green) up to the g (lime) and e subunits (dark green). This would lead to the removal of lipids within the c-ring (cyan) and the displacement of the F1 sector from the Fo, leading to mPTP opening.

F-ATP synthase (Figure 3) is a ~600 kDa multi-subunit enzyme that comprises two moieties, the membrane-embedded Fo sector, and the catalytic F1 sector, which are connected by two stalks, namely the peripheral stalk, which is structurally part of the Fo moiety, and the central stalk, which is related to the F1 sector. It should be noted that F-ATP synthase forms dimers within the inner mitochondrial membrane [89] via Fo-Fo interactions [90,91] that organize into long rows of oligomers that maintain the typical *cristae* morphology [92].

The globular evolutionary-conserved F1 sector is composed of 3 α- and 3 β-subunits that alternate in surrounding the central stalk, which in Mammals comprises subunits γ, δ, and ε [89]. According to Boyer’s catalysis model [93], the Fo sector allows the flow of H^+^ down their electrochemical gradient generated by the respiratory complexes across the membrane [94]. This induces the rotation of the c-ring that in turn generates a torque of the central stalk transmitted to the F1 sector where the ATP synthesis occurs [94]. Subunit γ rotation within α_3_β_3_ takes each of the three β subunits through the conformations βDP (bound to ADP), βTP (bound to ATP), βE (empty) thereby synthesizing three Mg^2+^-ATP molecules during each 360° rotation [95,96]. F-ATP synthase is a reversible rotary machinery able to hydrolyze ATP generating a pmf [89]. Both in the synthesis and in the hydrolysis directions, Mg^2+^ is essential for catalysis, and it can be replaced by other divalent cations, such as Mn^2+^ and Co^2+^ [97,98]. Intriguingly, Ca^2+^ ions only sustain ATP hydrolysis by F1 which is associated with proton translocation [99] and not coupled with the generation of pmf neither in Bacteria [98] nor in Mammals [97].

In humans [100], the Fo sector comprises 8 copies of subunit c, which is arranged in a circular structure (c-ring), and subunits a, A6L (ATP8), e, f, g, DAPIT(k) and 6.8PL(j). Subunits a and c form two semi-channels that allow the H^+^ flow by protonation/deprotonation reactions which involve conserved amino acid residues [94]. The cryo-EM structure of mammalian F-ATP synthase recently resolved by Sazanov’s group [101] revealed that subunit e, located at the basis of the peripheral stalk, is structurally linked to the lipid plug at the center of the c-ring. The mammalian peripheral stalk comprises subunits b, F6, d, and OSCP, and it contacts subunits a, A6L, f, e, and g of the membrane-embedded part of the Fo sector [102,103].

Structural and functional coupling of the Fo and F1 sectors is guaranteed by the OSCP subunit, since it prevents the co-rotation of the α_3_β_3_ subcomplex with the γ subunit during the proton flow, thereby ensuring enzyme efficiency [103]. Moreover, this coupling ensures enzyme sensibility to oligomycin, an antibiotic produced by *Streptomycetes* which binds the c-ring preventing its rotation [104], which is widely used in experimental characterization of mitochondria and F-ATP synthase.

The first clue that the F-ATP synthase could be involved in mPTP formation was the demonstration that CyPD interacts with the OSCP subunit of the F-ATP synthase peripheral stalk [26,27]. CyPD binding required relatively high concentrations (10 mM) of Pi, which probably acts by charge neutralization favoring the electrostatic interactions between the two proteins, and resulted in partial inhibition of ATP synthesis and hydrolysis; CsA dissociated CyPD from the F-ATP synthase removing this inhibitory effect [26]. A few years later clear evidence was obtained that a Ca^2+^-dependent channel can form from F-ATP synthase [27,105], as now supported by a variety of electrophysiological studies using highly purified enzyme preparations from bovine [28] and porcine [29] hearts. Based on extensive mutagenesis studies [106,107,108,109,110,111,112] and structural work [101] the “death finger model” (Figure 3) has been proposed as a plausible mechanism through which F-ATP synthase could generate a channel [43,113,114]. According to this model, binding of Ca^2+^ at the catalytic metal site located at the α/β subunits interface [106] causes a spatial rearrangement of the F1 sector that is transmitted from the OSCP subunit to the peripheral stalk at the point of entrance into the membrane, where subunit b forms a hairpin tightly associated with the C- and N-termini of subunits g and e, respectively. The C-terminus of subunit e, which contacts the c-ring, exerts a pulling effect on the lipids allowing formation of a channel within the c-ring by displacing the outer lipid plug. Binding of CyPD or of its chemical mimic benzodiazepine (Bz)-423 [27] favors the Ca^2+^-induced mechanical stress on OSCP, overcoming its ‘shock absorber’ function during rotation of subunit c.

Recently it has been observed that the synthetic c subunit of F-ATP synthase under high calcium concentrations adopts a cross-β conformation with channel properties influenced by CyPD [115,116]. This led to suggest an alternative but not mutually exclusive mechanism of mPTP formation in the context of neurodegenerative diseases that involves the participation of the c subunit together with mitochondrially targeted amyloid peptides Aβ and α-synuclein [117], whose involvement in pathology will be described in the following chapters of this review.

The role of the PPIase activity of CypD on the PTP modulation is still debated. It was originally suggested that CypD activates the PTP independent of its PPIase activity [118]. On the other hand, *Ppif*^−^/^−^ fibroblasts were protected against oxidative stress, and this protection was lost in cells expressing a wild-type CypD but not in cells expressing the inactive R96G CypD, suggesting that the PPIase activity of CypD facilitates PTP activation [72]. However, the exact mechanism is unknown. Future studies will be needed to address the role of the PPIase activity in regulating the PTP. Interestingly, in a recent study the CyPD PPIase activity, measured in different tissues including the brain, correlates with an increased ATP synthasome assembly (an ATP synthase supercomplex containing ANT and the phosphate carrier) and the formation of high-order ATP synthase oligomers, decreasing the probability of PTP formation [39].

More details on mPTP can be found in recent specific reviews [43,119].

## 7. Post-Translational Modifications of CyPD

**Figure 4:**
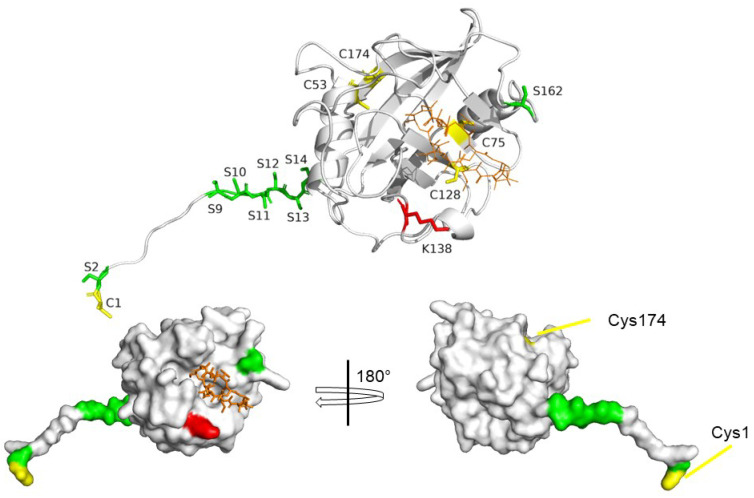
Post-Translational Modification of CyPD. Cartoon (upper panel) and surface (lower panel) representation of CyPD (AlphaFold structure) active site side (upper panel, lower left panel) and back face side (lower right panel) and PTM target sites. Red: acetylation site. Yellow: cysteine residues. Green: serine residues involved in protein phosphorylation.

Post-translational modification (PTM) of proteins dramatically expands the cellular proteome and enables rapid responses to changes in the biochemical environment and fine-tuning of cellular metabolism [120]. Here, we will describe the principal PTMs that affect CyPD and its function within the mitochondrial proteome, i.e., acetylation, phosphorylation, and oxidation (Figure 4). These studies have mainly been performed in the heart [121,122,123,124,125,126] and liver tissues [127] and/or in cellular models [31,32,128], while very few data on CyPD PTMs have been obtained in the brain and especially in neurodegenerative disorders [129], a gap that would worthwhile to be filled.

### 7.1. CyPD Acetylation

Protein acetylation refers to the transfer of the acetyl group of an acetyl coenzyme A (Ac-CoA) molecule to either the N-terminus of the polypeptide chain or the ε-amino group of lysine residues, which reduces the positive charge of the protein target [130]. The removal of the Ac-CoA from the protein is exquisitely enzymatic, being mediated by protein deacetylases. Mitochondrial deacetylases comprise three NAD+-dependent sirtuins, i.e., SIRT3, SIRT4 and SIRT5 [131]. While SIRT3, has the most robust deacetylase activity [132], SIRT4 and SIRT5 are thought to contribute to mitochondrial deacetylation to a lesser extent [42].

Lysine acetylation has been shown to be a widespread modification in the mitochondrial proteome: about 20% of mammalian liver [133] and heart [42] proteins have been found acetylated at a basal level, and more than 60% of the mitochondrial proteins contain acetylation sites [132]. Intriguingly, it has been demonstrated that CyPD removal alters the cardiac mitochondrial acetylome [42].

CyPD itself is a target of acetylation, in particular at the highly conserved Lys138 (*K167* in human/*K166* in murine) [121]. It has been demonstrated both in vitro and in vivo that acetylated CyPD is a substrate of SIRT3, and that CyPD acetylation is associated with an increase in mPTP opening [121,122,123,127,129]. The acetylated Lys138 is located in the catalytic site of CyPD, which is thought to be involved in the interaction with OSCP [27]. This interaction is sensitive to salts [27] at a concentration similar to the physiological one [134], suggesting that the acetylation of Lys138 enhances the interaction between CyPD and OSCP by lowering the sensitivity of the CyPD/OSCP complex to ionic strength. These findings are in line with the observation of a highly positively charged OSCP surface (pI = 9.97) in the F-ATP synthase structure [101].

### 7.2. CyPD Phosphorylation

Protein phosphorylation [135] involves the covalent introduction of a dianionic phosphate group to specific residues, a process that is mediated by protein kinases [136] and is reversed by protein phosphatases [137].

Different CyPD phosphoforms have been proposed [31,32,124,125,127]. While it is clear that the different phosphoforms have different effects on mPTP opening, much research is still needed to thoroughly characterize them all. In particular, it has been shown that CyPD is a substate of glycogen synthase kinase 3β (GSK-3β) [31], which phosphorylates CyPD and sensitizes mPTP opening [31,127,138]. It has been independently shown that Ser162 (*S191*) phosphorylation increased undoubtedly both the binding of CyPD to the OSCP subunit of F-ATP synthase and mPTP opening [124]. Intriguingly, the authors found that mutating the stretch of six serine residues at the N-terminus Ser9-14 (*S38-43*) into a stretch of six alanine residues increased the global phosphorylation levels of CyPD, probably at Ser162 (*S191*) and decrease mPTP propensity to open, suggesting that Ser9-14 may act as a negative regulator of the phosphorylation status of CyPD [124]. Similarly to the acetylated Lys138 (K166), Ser162 (S191) is located to the CsA-binding site side of CyPD, i.e., at the putative OSCP interacting surface. Both serine phosphorylation and lysine acetylation lower the positive charge of the respective target residues, suggesting that these two modifications co-operate to strengthen the interaction with OSCP by changing the electrostatic surface of the interaction site. Moreover, in silico studies suggested that both the phosphorylation of Ser162 (*S191*) and the acetylation of Lys138 (K166) affects the conformational ensemble of CyPD [139].

On the other hand, it has been reported that the germline deletion of the mitochondrial calcium uniporter (MCU) resulted in an increase in phosphorylation of CyPD at Ser13 (*S42*), and that this was associated both with an increase in CyPD binding to F-ATP synthase and mPTP propensity to open in heart cells [125]. These results seem in apparent discrepancy with those reported by Hurst et al. [124] and are difficult to rationalize on structural bases, as Ser13 is located far from the catalytic site. It is noticed, however, that: (i) the immunoprecipitation experiments were performed on the complete F-ATP synthase by Parks et al. [125], and on a single OSCP subunit by Hurst et al. [124], then one could hypothesize that phosphorylation of CyPD at Ser13 could also mediate its interaction towards another subunit of F-ATP Synthase (yet to be identified) other than OSCP, thus increasing the sensitization of mPTP opening; (ii) all the experiments performed by Parks et al. were carried out on murine cells, in which there are only three serine residues in tandem, while in human CyPD there are six (Ser9-14), thus suggesting that the regulation of phosphoCyPD-dependent mPTP opening in murine CyPD and human CyPD could be slightly yet at the same time strikingly different. In both cases, the N-term of CyPD appears to play a critical role.

It has also been reported that in patient-derived tumors phosphorylation of the N-terminal Ser2 by the kinase Akt2 has been associated with resistance to cell death [32]. Similarly to Ser9-14, the serine found in this work is structurally far from the active site of CyPD: it would be worth investigating how a point mutation at this site could lead to such a dramatic effect as the loss of enzymatic activity [32].

Altogether, it is clear that multiple phosphoforms of CyPD exist and that each has a different effect on the regulation of mPTP opening. While it was shown that CyPD is a target of at least two cytosolic kinases, i.e., GSK-3β [31] and Akt [32], that under specific conditions translocate within the mitochondrial matrix, there are still some fundamental data missing, such as: (i) what are the actual phosphoforms of CyPD, i.e., could multiple serine/threonine residues be phosphorylated at the same time? (ii) What is the stoichiometry of the different phosphoforms of CyPD? (iii) How can the N-terminal serine residues affect CyPD phosphorylation status and its regulation of the mPTP? (iv) What are the mechanisms that induce the translocation of cytosolic kinases within the mitochondrial matrix?

### 7.3. Oxidative Modifications of CyPD

A number of PTMs involve the chemical modification of proteins mainly at the level of cysteine residues [140] due to the reaction of their thiol group with reactive oxygen species (ROS), which include hydrogen peroxide, superoxide anion and the hydroxyl radical, and reactive nitrogen species (RNS), which include nitric oxide and its derivative peroxynitrite [141]. Mitochondria are the prime contributor of both ROS [142] and peroxynitrite [143].

The oxidative modification of CyPD has been investigated in the literature, with a particular focus on cysteine residues [126,128,144,145]. Of the five cysteine residues in the mature CyPD, Cys1 (*C30*) and Cys128 (*C157*) are solvent-exposed, while Cys53 (*C82*), Cys75 (*C104*), and Cys174 (*C203*) are more buried (Figure 4). According to the CyPD crystal structure [53,54], no disulfide bonds are observed, although the pairs Cys53-Cys174, located on the back face of the protein, and Cys75-Cys128 on the opposite side of the protein are close to each other. A closer analysis of the X-ray structure reveals that the first cysteine pair also displays a rather favorable side-chain geometry for S-S formation. It was Linard et al. who first reported that CyPD could be oxidized, that upon oxidation PPIAse activity is reduced by 20% and that Cys174 (*C203*) was the oxidation site [144]. The authors also suggested the formation of the Cys128-Cys174 disulfide bridge, but they could not detect it via mass spectrometry. Indeed, such a disulfide bridge would entail dramatic structural rearrangements of the protein which are quite unlikely to happen, especially considering that both Cys174 and Cys128 have other proximal cysteine residues and that such a conformational reorganization unlikely would retain the PPIase activity.

The role of the oxidative modifications of Cys174 (C203, C202 in murine models) on CyPD function, particularly on mPTP regulation, has been extensively investigated by Murphy’s group [126,128]. They found that treatment with a nitric oxide donor (GSNO) or mutation of Cys174 into a serine-desensitized H_2_O_2_-induced mPTP opening [128]. Moreover, the C174S mutants display a decreased binding on F-ATP synthase upon oxidation, compared to the wild-type counterpart [126]. Given that treatment with GSNO was effective only in wild-type cells, they concluded that Cys174 can be S-nitrosylated and that this PTM inhibits mPTP opening, as observed in ischemic preconditioning experiments [146]. The same group found that Cys174 (C203) is also a site of S-acylation and that the removal of the acyl group is necessary for mPTP opening [126], indicating the integration of multiple signals that converge on the modification of Cys174. Indeed, it has also been found that CyPD is part of the mitochondrial thioredoxin system [145], which indicates that CyPD oxidation is finely tuned within the mitochondrial matrix.

To conclude, also in the case of oxidative PTMs, there are several questions waiting to be answered. In particular, the structural modifications upon Cys174 (C203) oxidation and its relation to the regulation of mPTP should be addressed.

## 8. CyPD in Brain

Although CyPD is ubiquitously expressed in human tissues, its expression levels are tightly regulated and vary significantly among different districts [147,148,149]. Indeed, it has been found that while liver [147,148,149] and heart [149] mitochondria undergo CsA-sensitive swelling upon calcium treatment, adult brain mitochondria are highly resistant to this phenomenon [147,148,149], although both liver and brain mitochondria are able to swell, similarly, upon treatment with the channel-forming peptide alamethicin [147]. It was demonstrated that brain mitochondria resistance to swelling depends on the reduced CyPD levels, which were found almost three times lower than in heart and liver mitochondria [149]. More in detail, CyPD expression levels were found downregulated in mature (3-months old) rats when compared to newborn (1-day old) rats [149], which was also associated with different propensities to CsA-sensitive mPTP opening (lower in 3-months mice than in 1-day mice) [149]. Moreover, neuronal precursor PC12 cells treated with the differentiation factor NGF showed a dramatic reduction in CyPD expression level, confirming its differentiation-dependent down-regulation in mature neural cells [149]. Independently, another group found that neural CyPD levels increase during aging in both mice and humans [150,151,152] and that this correlates with calcium-handling capacity [150]. It should also be noted that *Ppif*^−^/^−^ mice showed neurological symptoms, i.e., an increase in anxiety-type behavior [153]. This could be both a direct or indirect effect of the absence of CyPD since they also had an abnormal accumulation of white adipose tissue [153] which has been shown to be associated with an increase in anxiety-type behavior [154].

## 9. The Involvement of CyPD in Alzheimer’s Disease

AD represents the most common form of dementia [155]. The progressive neuronal and synaptic loss typical of AD has two prominent pathological features, i.e., the abnormal accumulation of amyloid β peptide (Aβ) and the phosphorylation of tau protein in the brain [155]. The neurotoxic Aβ peptide is the product of amyloid precursor protein (APP), a transmembrane protein, cleaved by β and γ secretase (presenilins) [156]. APP cleavage products form the pathological amyloid plaques [157]. According to the “amyloid cascade hypothesis” [158], Aβ accumulation is the primary cause of AD. While at first the emphasis was put on the toxicity of extracellular amyloid plaques, the attention was then switched to the pathological intracellular events associated with Aβ that ultimately resulted in plaque formation [157]. Indeed, it has been found that Aβ accumulates in several subcellular compartments, including mitochondria [159], which is associated with a dampening of cellular respiration [160] and an increase in oxidative stress [161]. Although the amyloid hypothesis has been challenged [162] particularly due to the failure of anti-Aβ therapies [163], Aβ-mediated cellular dysfunction is still a relevant feature of the AD pathology. Its presence in non-AD individuals [164] suggests its possible involvement in the very first subtle steps of AD pathogenesis. On the other hand, tau is a microtubule-binding protein usually found in axonal and dendritic microtubules [155,165]. Hyperphosphorylation of tau, a hallmark of AD and other neurological pathologies, induces its re-localization within the neuron and its aggregation into the toxic neurofibrillary tangles [155,165].

It has been found that CyPD and Aβ interact within AD brain mitochondria. Surface plasmon resonance analysis revealed a specific interaction between recombinant Aβ(1-42) and CyPD in the nanomolar range (164 nM), which was even higher in the case of Aβ(1-42) oligomers (4 nM) [150]. CO-IP and immunochemistry experiments confirmed the presence of this interaction in the AD brains of both transgenic animal models and human patients [150,166]. The structural features of these interactions have not been described, i.e., the CsA-sensitivity of the interaction has not been tested, so it is not possible to conclude whether it occurs on the active site side or the back face of CyPD. Indeed, from this point of view, only an in silico prediction of the interaction between CyPD and Aβ has been made, but the conclusions were only drawn from a subcellular localization and an energetic point of view [167].

Nevertheless, the pathophysiological consequences of the CyPD/Aβ interaction have been thoroughly investigated. The age-associated increase in CyPD expression levels observed in the brain have been found exacerbated in the context of AD, specifically in an Aβ-rich context, as it was observed in neurons in the temporal cortex and hippocampus neurons but not in the cerebellum [150,166]. Specifically, elevated Aβ levels are associated with an age-dependent dysregulation in calcium handling, an increase in mitochondrial swelling, mPTP opening, and cytochrome c release, all of which are reversed by CsA treatment or CyPD knockout [150,152,166]. Intriguingly, *Ppif*^−^/^−^ mAPP mice, i.e., murine models of AD [168], exhibited a strong learning and spatial memory capacity than mAPP mice [150]. Indeed, the PKA/CREB signaling pathway, which is associated with synaptic plasticity [169], was found impaired in a CyPD/Aβ-dependent manner, and pharmacological or genetic inhibition of CyPD restored the axis activity in the context of AD [170]. Intriguingly, it has also been shown that CyPD levels decrease with the knock-out of tau and increase with its overexpression [171], strengthening the link between mPTP opening and the development of AD.

While brain CyPD levels steadily increase with age, the OSCP subunit of F-ATP synthase follows the exact opposite trend [152,172]. The reduction in OSCP levels, which is specific and does not concern other F-ATP synthase subunits, is also observed in the context of AD [172], which intriguingly has also been observed in AD patient-derived fibroblasts [173]. OSCP knock-down is associated with an increase of CsA-independent mPTP opening [172], consistent with the findings of other research groups [110], which is probably due to the opening of ANT-mediated mPTP [110]. Moreover, it has been found that Aβ interacts with OSCP [172] and that OSCP/CyPD interaction increases during aging [152], which is once again exacerbated in the context of AD [174].

The specific reduction in OSCP levels suggests a post-translational degradation of the protein since (i) OSCP knockdown also induces changes in the expression of other F-ATP synthase subunits [110] and (ii) the increase in CyPD expression was associated with an increase of OSCP ubiquitination [174], although other degradation pathways could be involved.

Despite the above studies reporting a detrimental effect of CyPD-mediated mPTP opening, it should be noted that it has also been associated with an important physiological role, i.e., the filopodiagenesis of neurons [175], which is impaired upon inhibition of CyPD. Similarly, it has been shown that an aberrant increase in SIRT3 expression, which promotes deacetylation of CyPD, hampers the mPTP flickering and is associated with motor impairment in a murine model of spastic paraplegia [129]. Moreover, CyPD knock-out impairs mPTP role in calcium handling, which was shown to lead to an increase of mitochondrial calcium and ultimately the activation of phospholipase A2, which induces the production of free fatty acids that impair oxidative phosphorylation and decreases the ATP levels [176]. Therefore, while CyPD inhibition has been shown to have ameliorative effects on the AD phenotype, it should be emphasized that it could also have detrimental effects on mitochondrial function, which should be preserved. Accordingly, two important pieces of information are missing. First, little attention has been put into characterizing the detailed structural features of the interaction between CyPD/Aβ/OSCP, which could be directly addressed with specific inhibitors without dramatically altering physiological mPTP opening and/or mitochondrial calcium handling. Second, CyPD PTMs in the context of AD have never been investigated. Specifically, we believe that both CyPD oxidation and CyPD acetylation could play a role in the development of AD, given that: (i) oxidative stress is a hallmark of AD [161,177] and, accordingly, treatment with antioxidants has been shown to be protective from Aβ-mediated insult similarly to genetic inhibition of CyPD [170]; (ii) aging and aging-associated diseases are characterized by a decline in NAD+ levels [178], which can lead to an inhibition of SIRT3 and an increase in CyPD acetylation [121], which induces mPTP opening [121]. Indeed, it has been shown that both brain mitochondrial acetylome and phosphoproteome are altered in AD [179,180], although little is known about the acetylation and phosphorylation status of CyPD in this compartment. Altogether, this would create a vicious cycle in which aberrant acetylated CyPD-dependent mPTP opening exacerbates oxidative stress, favoring oxidation of CyPD which, in turn, increases long-lasting mPTP opening. The investigation of these aspects could offer new insight into the participation of CyPD in the pathogenesis of AD.

## 10. The Involvement of CyPD in Parkinson’s Disease

Parkinson’s disease (PD) is the second most common neurodegenerative disease characterized by a chronically progressive, age-related, fatally invalidating movement disorder. Although several neuronal types are affected, dopaminergic neurons in substantia nigra pars compacta (SN) are among the first neurons to degenerate, leading to dopamine depletion in the striatum and motor symptoms [2]. The vast majority of PD cases are considered idiopathic, for which the greatest risk factor is aging, as demonstrated by epidemiological quantitative evidence showing a closely linked connection between aging and PD [181]. Hallmarks of PD include the misfolding and aggregation of α-synuclein (α-Syn) into intracytoplasmic protein deposits known as “Levy bodies” (LB), oxidative stress leading to oxidized dopamine, mitochondrial dysfunction, and inflammation, all features that are facilitated by the age-related metabolic alterations [2,182,183].

Despite the great number of studies, the molecular mechanisms underlying dopaminergic neuronal death are still debated. As LB or an earlier oligomeric form of α-Syn are thought to be toxic [184,185] and able to spread in synaptically coupled brain networks [186,187], it has been proposed that LB triggers neuronal dysfunction and death. Consistently, a point mutation in the *SNCA* gene encoding α-Syn or duplication/triplication of *SNCA* increases the risk of developing PD [188,189]. However, post-mortem analysis of human brains showed that the correlation between LB and neuronal death is weak [183].

An alternative (and not mutually exclusive) hypothesis is that the loss of dopaminergic neurons is driven by mitochondrial dysfunction, leading to bioenergetic failure, oxidative stress, increased mtDNA damage, and mitophagy defects [182]. Mitochondria became suspects in PD etiology by the discovery that complex I activity is reduced in the SN [190] and skeletal muscle [191] of PD cases. Moreover, treatment of dopaminergic neurons with complex I inhibitors, notably 1-methyl-4-phenyl-1,2,3,4-tetrahydropyridine (MPTP) and the pesticide rotenone, leads to increased ROS production [192], and activation of the NLRP3 inflammasome, which triggers the subsequent secretion of interleukin 1β [193], ultimately resulting in neuronal damage. Indeed, the use of these inhibitors is the basis of several animal and cellular models of PD. Consistently with these observations, a recent study demonstrated that selective disruption of complex I in mouse dopaminergic neurons by intersectional genetics was sufficient to cause progressive, human-type parkinsonism [194].

Another piece of evidence for a major contribution of mitochondrial dysfunction to motor deficits comes from studies of familial cases of PD. Recessive, early-onset forms of PD are linked to loss of function mutations in three gene products that are directly involved in mitochondrial biology, i.e., PINK1, encoding a mitochondria-targeted kinase, and Parkin/PARK2, encoding an E3 ubiquitin ligase, both involved in mitophagy and mitochondrial biogenesis and DJ-1, encoding a redox-regulated chaperone essential for oxidant defenses [2]. For example, in dopaminergic neurons derived from a patient with a homozygous mutation in DJ-1, mitochondrial oxidative stress triggers a pathogenic cascade that ultimately results in lysosomal dysfunction and α-Syn accumulation [195]. Mutations in genes associated with dominant forms of PD have also been linked to mitochondrial dysfunction. These include: (i) SNCA, encoding α-Syn [196,197], (ii) GBA, encoding the lysosomal glucosylceramidase β, which participates in glycolipid metabolism [198], and (iii) LRRK2, a kinase associated to the outer mitochondrial membrane that is involved in mitophagy [199].

Dopaminergic neurons in the SN are considered more vulnerable to mitochondrial dysfunction than other neuronal cells, due to their morphology (long and highly branched axon) and distinctive physiology, characterized by high and slow oscillations in intracellular calcium concentration triggered by the opening of the plasma membrane Cav1 calcium channels and the release of calcium from endoplasmic reticulum stores [183]. Different major factors underlying declining mitochondrial function have been proposed. According to one hypothesis [183], the typical calcium oscillations promote calcium entry into mitochondria at specialized junctions with the endoplasmic reticulum (MAMs) [200], stimulating the OXPHOS activity in the absence of strong ATP demand and leading to mitochondrial hyperpolarization, which causes an increase of ROS production and diminishes the autophagic capacity of neurons and their ability to deal with misfolded α-Syn fibrils [201]. However, in a recent clinical trial, a limited effect of the L-type channel inhibitor dihydropyridine isradipine was observed in newly diagnosed patients with PD [202], despite in dopaminergic neurons this drug reduced oxidative stress, mitochondrial and lysosomal dysfunctions, and α-Syn toxicity [203,204].

The involvement of the mPTP in the PD mechanisms has also been proposed, particularly its long-lasting opening, which causes mitochondrial depolarization, blocking of ATP production and mitochondrial swelling, and ultimately cell death [43]. Indeed, complex I toxins that increase ROS production also activate mPTP [205,206], while the inactivation of CyPD is beneficial in preserving mitochondrial bioenergetic in an acute insult model of MPTP-induced dopaminergic neurotoxicity [207]. Moreover, a solid piece of evidence in favor of the mPTP as a major contributor to PD pathogenesis comes from a study showing that genetic ablation of CyPD in PD-linked α-Syn mutant transgenic mice delayed the disease onset and extended lifespan, thus providing a demonstration that CyPD-induced mPTP regulates the PD development [208]. Interestingly, a recent study reveals that neurons bearing a PD-linked mutation in *LRRK2* [209] or *GBA* [198] genes show NAD+ shortage, possibly as a consequence of the elevated activity of NADase SARM1, which has been observed to be reduced in neuronal cells from PD patients [210]. As observed above, NAD+ deficit could impair SIRT3 activity [211], thus increasing CyPD acetylation, which in turn is more prone to activate the mPTP [121], a hypothesis that has not yet been tested in animal or cellular models of PD. On the other hand, in vitro NMR experiments demonstrated that a truncated form of recombinant CyPD interacts directly with the proline-rich C-terminal domain of the soluble α-Syn, preventing its aggregation, as well as with preformed α-Syn fibrils favoring their disassembly, apparently mediated by the CyPD enzymatic activity [212]. While preventing protein disaggregation can be viewed as advantageous, disaggregation can produce small oligomeric species, whose toxicity is still debated, especially at the mitochondria level [213]. Further investigations are needed to evaluate whether this role of CyPD plays beneficial cellular effects.

It was reported that in neuronal cells treated with exogenous α-Syn a CsA-sensitive mPTP overactivation is triggered by the transition of α-Syn from its monomeric to oligomeric structure, probably as a consequence of a selective oxidation of the F-ATP synthase β subunit. Consistently, the threshold of mPTP opening by calcium ionophore was found lower in neuronal cells bearing *SNCA* triplication [197,214]. In familiar forms of PD, propagation of mPTP opening could also be favored by the inhibition of mitophagy, leading to the accumulation of fragmented mitochondria and failure in removing activated mPTP, which in turn increases ROS production [181]. Interestingly, in primary mouse cultured cerebral cortical neurons, it was demonstrated that activated mPTP contributes to neuronal Ca^2+^ overload by releasing Ca^2+^ into the cytoplasm during acute oxidative stress, while genetic ablation of CyPD reverses such release, leading to attenuation of the ATP decrease, and a significantly higher viability [215]. In accordance with these findings, a recent study showed that changes in membrane potential in peripheral blood mononuclear cells may function as an early indicator of neuronal death in PD, enabling earlier diagnosis [216].

As there is currently no disease-modifying treatment for PD, these studies suggest that mPTP may be a target for therapies. Efforts to identify mPTP inhibitors that are clinically useful are ongoing and very promising, as potent and metabolically stable inhibitors with an in vitro potency in the nanomolar range have recently been identified [217]. Nevertheless, as discussed by Rottenberg et al. [181], drugs, or other manipulations, should only inhibit damaging, long-lasting full openings of mPTP and not its short, partial openings that can be beneficial [129,175]. Interestingly, melatonin, which is known to have a protective effect against aging and neurodegeneration [218], has been shown to inhibit not transient [219], but only long-lasting mPTP openings [220]. Such a difference suggests that this latter inhibition could occur through a still unknown indirect mechanism. It is tempting to hypothesize that the observed melatonin interaction with SIRT3 [221] could also result in the modulation of CyPD acetylation and then mPTP opening [121], an effect still to be tested.

In conclusion, the emerging evidence demonstrates that mitochondrial dysfunction notably contributes to the development of PD and that CyPD modulation of mPTP opening plays a major role. CyPD itself may be a promising drug target, for which several inhibitors have been recently obtained [66], as discussed in the following section.

## 11. Pharmacological Inhibition of CyPD

Due to the established involvement of CyPD in various pathological conditions, much effort has been put into the development of small molecules able to inhibit its activity. It should be noted that the involvement of CyPD in pathology is almost always associated with its role in the regulation of the mPTP, for which there is still no detailed molecular structure [43]. While it is known that CsA desensitizes pore opening [45], it has not been fully established if only the CsA binding domain (i.e., the active site) of CyPD participates in mPTP regulation. As noted above, different studies revealed that several PTMs affecting mPTP regulation take place on the back face of CyPD [32,125,126,128], making it impossible to fully exclude its involvement in the binding of CyPD on mPTP. Therefore, while multiple, different binding sites of CyPD on mPTP could exist, all the research on CyPD inhibitors has focused solely on molecules binding to its active site.

The most significant challenge when it comes to the development of CyPD inhibitors is the molecular specificity. Indeed, the human genome encodes for at least 17 different CyPs (Table 1) with high homology to one another. According to Davis et al., the sequence identity between the PPIAse domains of different CyPs ranges from 61% to 86%, and the RMSD across all atoms is less than 2 Å, which lowers to 0.4–1.0 Å when the two most divergent structures (PPIL1 and PPWD1) are excluded [52]. Although several members of the CyP family have been poorly (if not at all) characterized and therefore their involvement in physiological processes has not been established, the development of isoform-specific inhibitors can greatly improve their safety profile by reducing the potential off-target effects. Particular relevance in this context is CyPA, which according to PaxDB is the most expressed CyP and one of the most abundant proteins in the cytoplasm [222].

Due to its immunosuppressant properties, non-immunosuppressive CsA-analogues have been synthesized. The simple addition of an isoleucine group at position 4 of CsA results in the non-immunosuppressive analogue N-methyl-4-isoleucine cyclosporin (NIM811) [223], which has been proposed for the treatment of hepatitis C virus infection [224]. The addition of the isoleucine group prevents the binding of the CyPA:CsA complex to calcineurin, abolishing the immunosuppressive properties of CsA. Another non-immunosuppressive CsA analogue is alisporivir (formerly Debio-025), which derives from the substitutions with an N-methylalanine and an N-ethylvaline at positions 3 and 4 respectively [225], which has also shown promising results in the treatment of patients co-infected by HCV and HIV [226]. CsA itself was found ineffective in patients with myocardial injury in phase III clinical trials [227], which raised the question of whether CyPD-dependent mPTP could be a relevant clinical target for such a condition [228].

CsA and its derivatives can be grouped into the general class of macrocyclic inhibitors, which also includes the macrolide sanglifehrin A (SfA), the cyclic decapeptide antanamide, and their analogues [229]. Macrocyclic inhibitors are however affected by several issues, including low selectivity over different CyPs and poor pharmacokinetic profiles [229]. Beyond the macrocyclic inhibitors, another numerous class of molecules comprehend the synthetic small-molecule inhibitors, which according to Haleckova et al. [229] can be further divided into:

(1) N-4-Aminobenzyl-N′-(2-(2-arylpyrrolidin)-2-oxoethyl)urea-based compounds,
(2) 2-(Benzyloxy)arylurea-based compounds
(3) Other small-molecule inhibitors

To date, the molecules in class I represent the most potent and best-characterized class of small-molecule CyPD inhibitors developed [229], although the issues of isoform-specificity and pharmacokinetic properties still stand. For instance, the inhibitor “C31” (i.e., 1-(4-aminobenzyl)-3-(2-(2-(2-(methylthio)phenyl)pyrrolidin-1-yl)-2-oxo-1-phenylethyl)urea), has been found to strongly inhibit CyPD PPIAse activity [230] and mPTP opening in isolated liver [230] and heart [231] mitochondria. Additionally, it has shown cytoprotective effects against I/R injury [230,231]. Notably, C31 retains its inhibitory effect on mPTP opening in *Ppif*^−^/^−^ mitochondria, suggesting a dual inhibition mechanism involving both CyPD-dependent and CyPD-independent pathways [230,231]. However, it should be noted that there were no differences in mPTP inhibition between wild-type and *Ppif*^−^/^−^ mitochondria [230,231], indicating that these two mechanisms, if they exist, do not have an additive or synergistic effect. Furthermore, while C31 demonstrated efficacy in protecting hepatocytes from I/R injury in vivo [230], no such protection was observed in the myocardium [231]. These findings underline the significance of testing the inhibitors on *Ppif*^−^/^−^ models to assess CyPD-specific effects and emphasize the need for extensive in vivo administration studies.

To improve the selectivity of CyPD-inhibitors, at least two strategies have been proposed. First, De Simone et al. suggested the development of tri-vector molecules, which not only bind to the active site of CyPD, targeted by all the inhibitors developed so far, but make also extensive contacts on the poorly conserved 3’o-clock pocket [58]. The second strategy was proposed recently by Peterson et al. [66] and it was shown to actually lead to a remarkable enhancement in CyP-selectivity. The authors exploited the S2 pocket to develop, through iterated molecule engineering guided by X-ray co-crystal structure, the first potent isoform-specific macrocyclic inhibitors of CyPD and CyPE [66]. The pharmacokinetic properties of these molecules have not been tested, but their striking selectivity indicates the importance of the underestimated S2 pocket in CyP-targeted drug discovery. This finding will likely pave the way for the synthesis of highly specific compounds, serving as a starting point to enhance their pharmacokinetic properties and have optimized drugs.

Beyond the obvious difficult task to have potent molecules suited from the pharmacokinetic point of view, when it comes to CyPD there are some major challenges in the drug discovery process. The sole fact that CyPD resides within the mitochondrial matrix represents a significant obstacle since its inhibitors need to overcome more physiological and physical barriers than drugs directed towards cytosolic targets. This aspect assumes particular significance within the context of the central nervous system (CNS), where CyPD represents an appealing target given its involvement in pathologies such as AD [150] and PD [208]. To reach the CNS, molecules must overcome a great physical obstacle, i.e., the blood-brain barrier (BBB), which, based on current research findings, has exhibited limited permeability to CyPD inhibitors [232,233]. One possibility to overcome this problem would be the involvement of liposomal drug delivery systems, which is a promising therapeutical approach [234] that, to our knowledge, has not been tested for CyPD inhibitors.

Despite the pharmacological inhibition of CyPD, we speculate that at least two other different strategies could be adopted. First, the characterization of CyPD PTMs in the context of AD and/or PD could lead to targeting not directly CyPD itself but rather its modifying enzymes, such as GSK3β [31,127] or SIRT3 [121,122]. Second, since the genetic inhibition of CyPD has been shown to have ameliorative effects on the development of mPTP-mediated cellular dysfunction, it is tempting to also suggest the employment of antisense oligonucleotides (ASOs) to knock-down its expression. Indeed, while the pharmacological inhibition of protein targets has the challenging task of developing specific and BBB-permeable molecules when it comes to ASOs one should keep in mind that (i) they are highly selective and much easier to be designed, (ii) they are already being employed in the treatment of several neurological diseases, such as spinal muscular atrophy and Duchenne muscular dystrophy [235] and (iii) much work is being put in the design of drug delivery across the BBB, for which there are already promising results [236].

## 12. Conclusions

**Figure 5:**
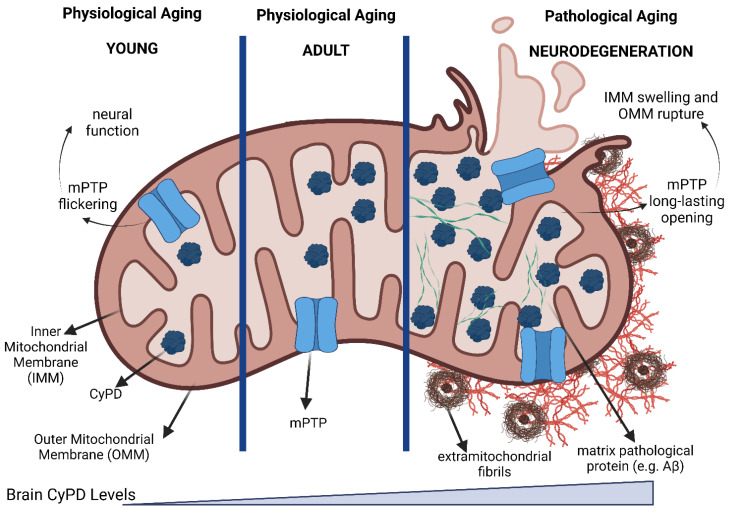
CyPD is a double-edged sword between health and pathology. During aging, CyPD levels in brain mitochondria steadily increase, which is associated with a different propensity of mPTP opening. In neurons, it has been shown that physiological mPTP activity has important effects, i.e., has been implicated in the neural filopodiagenesis. In the context of neurodegeneration, such as AD or PD, mitochondria are enriched of both CyPD and pathological proteins (such as Aβ, phosphorylated Tau, and α-Syn), which is associated with the aberrant mPTP opening that leads to both mitochondrial swellings, outer mitochondrial membrane rupture and, ultimately, cell death. Created with BioRender.com (accessed on 4 July 2023).

Neurodegenerative diseases (ND) represent a dramatic health and social burden that due to their molecular and biological complexity are still lacking an appropriate eradicative strategy. Aging remains a major risk factor for the development of various NDs, including AD and PD. The involvement of mitochondrial dysfunction in both these contexts has been extensively and convincingly documented, but many questions remain. CyPD has been shown to be almost absent in young brain mitochondria and to steadily increase with aging, which in turn is associated with a higher propensity for mPTP opening (Figure 5). In the context of AD-related mitochondrial dysfunction, CyPD levels are dramatically higher, and its inhibition has been shown protective at both the cellular and in vivo levels. It is tempting to speculate that aberrant CyPD-dependent mPTP opening may be a hallmark of ND-related mitochondrial dysfunction, which has been demonstrated for AD. However, it is important to note that mPTP also has physiological functions, as its flickering activity has been implicated in neuronal filopodiagenesis. Therefore, the complete inhibition of either mPTP or CyPD should not be the only focus of CyPD-targeted approaches, but rather fine-tuning its molecular function, such as reducing its abundance within mitochondria (through gene therapy) or modulating the enzymes responsible for its post-translational modifications. The complete ND-related picture of (i) CyPD levels, (ii) CyPD interactors, and (iii) CyPD PTMs could potentially provide novel CyPD-related molecular targets and offer a different perspective for the treatment of such pathologies.

## Figures and Tables

**Table 2:** Numbering of CyPD. Due to the differences between the gene product of *ppif*, the mature CyPD, and the crystal structures of CyPD deposited in the PDB, the same residue can be numbered in at least three different ways. Here, we will mostly consider the numbering of the mature CyPD, i.e., without the mitochondrial targeting sequence (MTS). The corresponding numbering of residue i in the immature CyPD, i.e., with the MTS, will be i + 29, while in the crystal structure will be i-13. The crystal structure numbering of CyPD is the same as the numbering of CyPA.

Mature CyPD	Immature CyPD (with MTS)	Crystal/CyPA
i	i + 29	i − 13

## Data Availability

Not applicable.
